# Supplementary material for: Mechanisms governing the pioneering and redistribution capabilities of the non-classical pioneer PU.1
Source: Nat Commun. 2020 Jan 21;11:402. doi: 10.1038/s41467-019-13960-2 (PMC6972792; doi:10.1038/s41467-019-13960-2)
Supplement: Supplementary file 7 — Source data [file 41467_2019_13960_MOESM7_ESM.zip › Source_Data/FigureS2/FigureS2B_MotifScanOutput/KmeansCluster14/homerResults/motif3.similar.html]

motif3

## Information for motif3

G
C
A
T
C
G
T
A
C
G
T
A
A
T
G
C
G
T
A
C
C
T
G
A
A
G
T
C
C
G
T
A
  
Reverse Opposite:  

C
G
A
T
A
C
T
G
A
G
C
T
A
C
T
G
A
T
C
G
A
C
G
T
G
C
A
T
C
G
T
A
  

|  |  |
| --- | --- |
| p-value: | 1e-48 |
| log p-value: | -1.126e+02 |
| Information Content per bp: | 1.858 |
| Number of Target Sequences with motif | 147.0 |
| Percentage of Target Sequences with motif | 28.82% |
| Number of Background Sequences with motif | 3151.1 |
| Percentage of Background Sequences with motif | 6.96% |
| Average Position of motif in Targets | 104.8 +/- 53.7bp |
| Average Position of motif in Background | 100.4 +/- 64.6bp |
| Strand Bias (log2 ratio + to - strand density) | -0.2 |
| Multiplicity (# of sites on avg that occur together) | 1.20 |
| Motif File: | file (matrix) reverse opposite |

### Similar de novo motifs found

|  |  |  |  |  |  |  |  |
| --- | --- | --- | --- | --- | --- | --- | --- |
| Rank | Match Score | Redundant Motif | P-value | log P-value | % of Targets | % of Background | Motif file |
| 1 | 0.947 | G C A T A T C G A G C T C T A G A T C G G A C T C A G T C A G T T G C A T A G C | 1e-48 | -111.432097 | 26.86% | 6.07% | motif file (matrix) |
| 2 | 0.958 | G A C T G C A T A T C G G C A T C T A G A C T G A C G T C G A T C G A T | 1e-43 | -100.348231 | 27.06% | 6.81% | motif file (matrix) |
| 3 | 0.918 | C G T A T G C A T A C G G T C A T C G A T C G A G T A C G T A C C T G A A T G C C G T A C T A G | 1e-42 | -97.729250 | 31.57% | 9.46% | motif file (matrix) |
| 4 | 0.979 | C G T A C G T A A G T C A G T C C T G A A G T C C G T A | 1e-41 | -95.376461 | 24.51% | 5.84% | motif file (matrix) |
| 5 | 0.937 | C T A G T A G C G T A C G C T A G C T A C T G A G T A C G T A C C T G A A T G C C G T A C T G A T A C G | 1e-40 | -94.379644 | 22.16% | 4.78% | motif file (matrix) |
| 6 | 0.955 | G C A T A C T G A G C T C T A G A T C G A G C T A C G T G C A T G T C A A T G C G A T C | 1e-40 | -94.362690 | 19.02% | 3.43% | motif file (matrix) |
| 7 | 0.860 | G A T C C G A T A T C G G A C T A C T G C T A G A G C T A C G T C A G T T C A G A T C G G C T A C T A G C T A G | 1e-26 | -60.583981 | 11.76% | 1.99% | motif file (matrix) |
| 8 | 0.853 | C A G T A C T G C G A T C T A G A C T G A G C T A C G T A G C T A G T C G A T C C G A T T A G C G A T C | 1e-24 | -56.439159 | 9.61% | 1.37% | motif file (matrix) |
| 9 | 0.858 | G A C T C A G T G T C A T G C A A T G C G T A C C G T A G A T C G C T A G C T A | 1e-20 | -47.590928 | 11.18% | 2.36% | motif file (matrix) |
